# SSW Library: An SIMD Smith-Waterman C/C++ Library for Use in Genomic Applications

**DOI:** 10.1371/journal.pone.0082138

**Published:** 2013-12-04

**Authors:** Mengyao Zhao, Wan-Ping Lee, Erik P. Garrison, Gabor T. Marth

**Affiliations:** Department of Biology, Boston College, Chestnut Hill, Massachusetts, United States of America; National Institutes of Health, United States of America

## Abstract

**Background:**

The Smith-Waterman algorithm, which produces the optimal pairwise alignment between two sequences, is frequently used as a key component of fast heuristic read mapping and variation detection tools for next-generation sequencing data. Though various fast Smith-Waterman implementations are developed, they are either designed as monolithic protein database searching tools, which do not return detailed alignment, or are embedded into other tools. These issues make reusing these efficient Smith-Waterman implementations impractical.

**Results:**

To facilitate easy integration of the fast Single-Instruction-Multiple-Data Smith-Waterman algorithm into third-party software, we wrote a C/C++ library, which extends Farrar’s Striped Smith-Waterman (SSW) to return alignment information in addition to the optimal Smith-Waterman score. In this library we developed a new method to generate the full optimal alignment results and a suboptimal score in linear space at little cost of efficiency. This improvement makes the fast Single-Instruction-Multiple-Data Smith-Waterman become really useful in genomic applications. SSW is available both as a C/C++ software library, as well as a stand-alone alignment tool at: https://github.com/mengyao/Complete-Striped-Smith-Waterman-Library.

**Conclusions:**

The SSW library has been used in the primary read mapping tool MOSAIK, the split-read mapping program SCISSORS, the MEI detector TANGRAM, and the read-overlap graph generation program RZMBLR. The speeds of the mentioned software are improved significantly by replacing their ordinary Smith-Waterman or banded Smith-Waterman module with the SSW Library.

## Introduction

The Smith-Waterman-Gotoh algorithm (SW) [1,2] is the most influential algorithm for aligning a pair of sequences. It is an essential component of the majority of aligners from the classical BLAST [3] to the more recent mappers. Although most of these aligners do not use SW directly to align a sequence to the whole genome sequence due to the quadratic time complexity of SW, they extensively use it for seed extension and for constructing the final alignment, and spend significant amount of CPU time on this algorithm. Due to the critical role of SW, many efforts have been made to accelerate SW, taking the advantages of special hardware such as single instruction multiple data (SIMD), field-programmable gate array (FPGA) and graphics processing unit (GPU) [4–6]. Among the three, SIMD based algorithms are most frequently used because they are compatible with most modern x86 CPUs. SIMD acceleration methods can be further divided into intra-sequence parallelization [7] and inter-sequence parallelization [8]. Inter-sequence parallelization is only useful when many pairs of sequencing reads are aligned simultaneously; intra-sequence parallelization however parallelizes for each single pairwise alignment, so it can be used more flexibly in various applications such as that needs aligning a single read against a potentially large genome reference sequence. Farrar’s Striped SW [9] with SWPS3’s [10] improvement is the fastest intra-sequence parallelized SIMD implementation running on x86 processors with the Streaming SIMD Extensions 2 (SSE2) instruction set. Indeed, Farrar’s algorithm has been embedded in several popular genomic sequence mapping tools, such as BWA-SW [11], Bowtie2 [12], Novoalign (http://www.novocraft.com/) and Stampy [13].

Though striped SW is tens of times faster than a standard SW implementation, only a few aligners have used this more advanced algorithm. There are several practical obstacles. Firstly, implementing a striped SW requires good understanding of SSE2 instructions and the more complex algorithm, which may take significant development time. Secondly, the original striped SW only gives the optimal alignment score but does not report the position or the detailed alignment, the information necessary for using SW as a component to construct the final alignment. How to report the position and alignment without affecting speed is non-trivial. Thirdly, while a few implementations report position and alignment, they are tightly integrated in a larger project and cannot be easily reused in other programs. Fourthly, when aligning a short read against a long sequence, we would like to know suboptimal alignments such that we can tell if the optimal position is trustworthy. Most existing libraries have not addressed this issue.

Although striped SW has been published for six years, we are still in lack of a fast, versatile and standalone library. This leads us to develop the SSW library, a light weighted but comprehensive C/C++ library for pairwise sequence alignment with the striped SW algorithm.

## Results and Discussion

### Implementation

To build a light-weight and easily reusable SIMD SW library for the genomic application development community, we made the SSW Library. It extends the Striped SW and SWPS3’s SIMD implementations to provide the mapping location and detailed alignment information (traceback), without performance penalty. Though these features are crucial when integrating SW into other genomic analysis systems, among the existing SIMD SW implementations only SSEARCH provides them, and as discussed in the Performance: Short-Read Genomic Alignment section its performance in typical genomic alignment contexts is poor. The SSW library can also return the heuristic suboptimal (second-best) alignment score and location without additional computational cost, which enables the use of the method in contexts that exploit this information in mapping-quality estimation. We describe our efficient implementation of these features in the Methods section.

### Usage

The SSW library is an application program interface (API) that can be used as a component of C/C++ software to perform optimal protein or genome sequence alignment. The library returns the SW score, alignment location and traceback of the optimal alignment, and the alignment score and location of the suboptimal alignment. We provide the library with an executable alignment tool that can be used directly to perform protein or DNA alignments. It is a demonstration of the API usage and a practical tool for accurate whole viral or bacterial genome alignment. Moreover, since this tool is sufficiently fast and memory-efficient for alignment to very large reference genome sequences, e.g. the human genome, it can also be used to validate alignments produced by heuristic read mappers. The instructions of how to install and run the library is described in the README file at the software website (https://github.com/mengyao/Complete-Striped-Smith-Waterman-Library). A test data set is also provided there. 

### Performance

#### Protein Database Search

We compared SSW’s performance (with and without returning the detailed alignment, SSW-C and SSW, respectively) to Farrar’s accelerated SW and SSEARCH (version 36.3.5c) on a Linux machine with 2GHz x86 64 AMD processors. We ran each program on a single thread. Since the optimal alignment scores for long DNA sequences given by SWPS3 are not consistent with others’, we did not benchmark its running time here.

**Figure 1:**
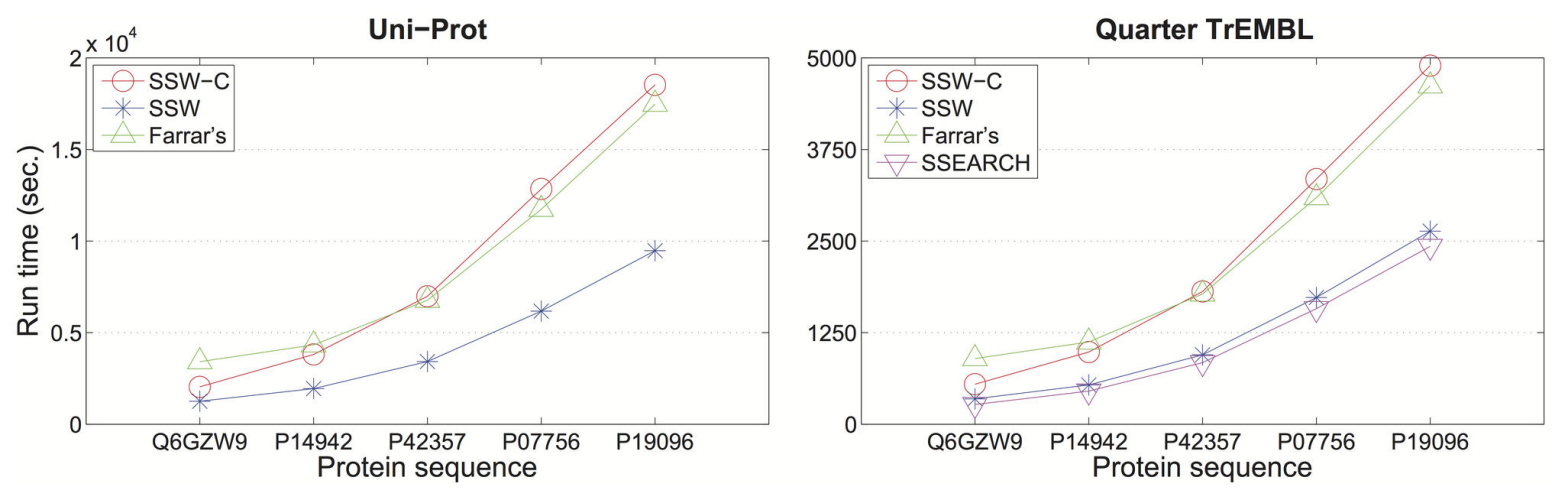
Running time of different SW implementations for protein database search. 5 query proteins were searched against the whole Uni-Prot database (left) and one quarter of the TrEMBL database (right). Running time is shown on the y-axis for SSW without (blue) and with (red) detailed alignment, Farrar’s implementation (green) and SSEARCH (pink). All SW implementations used the BLOSUM50 scoring matrix with gap open penalty -12 and extension penalty -2.

To measure the speed of protein database searching, we aligned five protein sequences (Q6GZW9 (75 aa), P14942 (192 aa), P42357 (551 aa), P07756 (1283 aa), and P19096 (2154 aa)) against the Uni-Prot Knowledgebase release 2013_09 (including Swiss-Prot and TrEMBL, a total of 13,823,121,038 aa residues in 43,362,837 sequences), by all four algorithms (see Figure 1). Since SSEARCH did not return alignment results against the entire Uni-Prot database, we were only able to test it against one quarter of the TrEMBL sequences (3,872,274,471 aa in 10,705,468 sequences). The command lines used for the database search are given in Table S1 in File S1. Our SSW algorithm is the fastest or equally fast to SSEARCH across the entire protein sequence length range we tested.

We also compared the CPU SW implementations with one of the most popular GPU implementations, CUDASW++ [4,5]. We were unable to obtain access to a system with a GPU card supporting CUDASW++ 3.0, so we ran CUDASW++ 2.0 (on a GeForce GTX 480 graphics card with 1.5G memory) for the comparison. Since our GPU host machine does not have sufficient storage for a larger protein database, such as TrEMBL, we aligned the five query proteins against the Swiss-Prot database (release 2013_09). The total running times in seconds of SSW, SSW-C, Farrar’s, SSEARCH, and CUDASW++ 2.0 are 310.10, 597.35, 424.27, 270.94 and 66.75 respectively. This GPU SW is about four fold faster than the fasted CPU SW. However, we did see the difficulty of using GPU SW, e.g. hardware is not easy found and installation is not ordinary.

#### Short-Read Genomic Alignment

**Figure 2:**
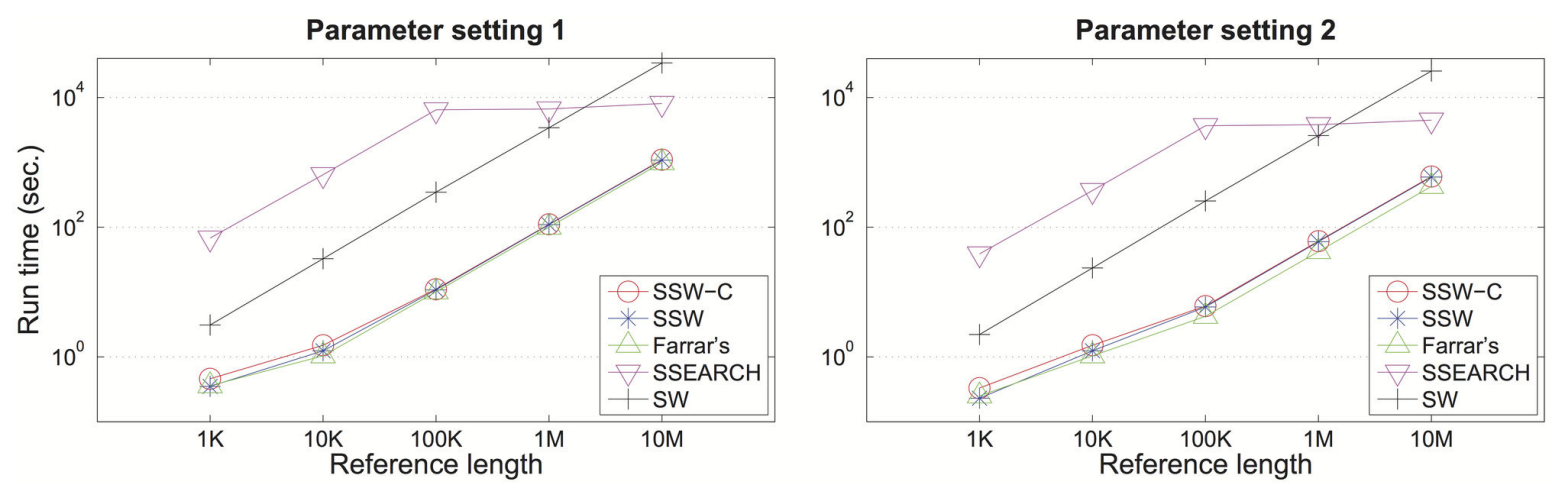
Running time of different SW implementations for simulated genomic read alignment. Running time of aligning 1,000 simulated Illumina reads to human reference sequences of various lengths. The log-scaled running time is shown on the y-axis for SSW without (blue) and with (red) detailed alignment, Farrar’s implementation (green), SSEARCH (pink) and an ordinary SW implementation (black). All SW implementations were tested under two sets of SW parameters: scores of match, mismatch, gap open and extension are 2, -1, -2, and -1 respectively (left), and scores of match, mismatch, gap open and extension are 1, -3, -5, and -2 respectively (right).

To benchmark genome sequence alignment, we tested the programs with both simulated data and real sequencing reads. We selected 1Kb - 10Mb regions from human genome chromosome 8, and using an Illumina read simulator (http://www.seqan.de/projects/mason/) we generated a thousand 100 bp-long sequences from these regions. We then aligned these reads back to their corresponding reference sequences with each of the five algorithms (including our naïve Smith-Waterman, https://github.com/wanpinglee/SmithWaterman) and compared their running times (see Figure 2). Each program was run on a single thread on a Linux machine with 2G MHz x86_64 AMD processors. Two SW parameter settings are employed in the experiments: setting 1 (scores of match, mismatch, gap open and extension are 2, -1, -2, and -1 respectively) and setting 2 (scores of match, mismatch, gap open and extension are 1, -3, -5, and -2 respectively). The command lines used for read alignments are shown in Table S2 in File S1. Our SSW algorithm and Farrar’s accelerated version are equally fast for the whole reference length spectrum.

**Figure 3:**
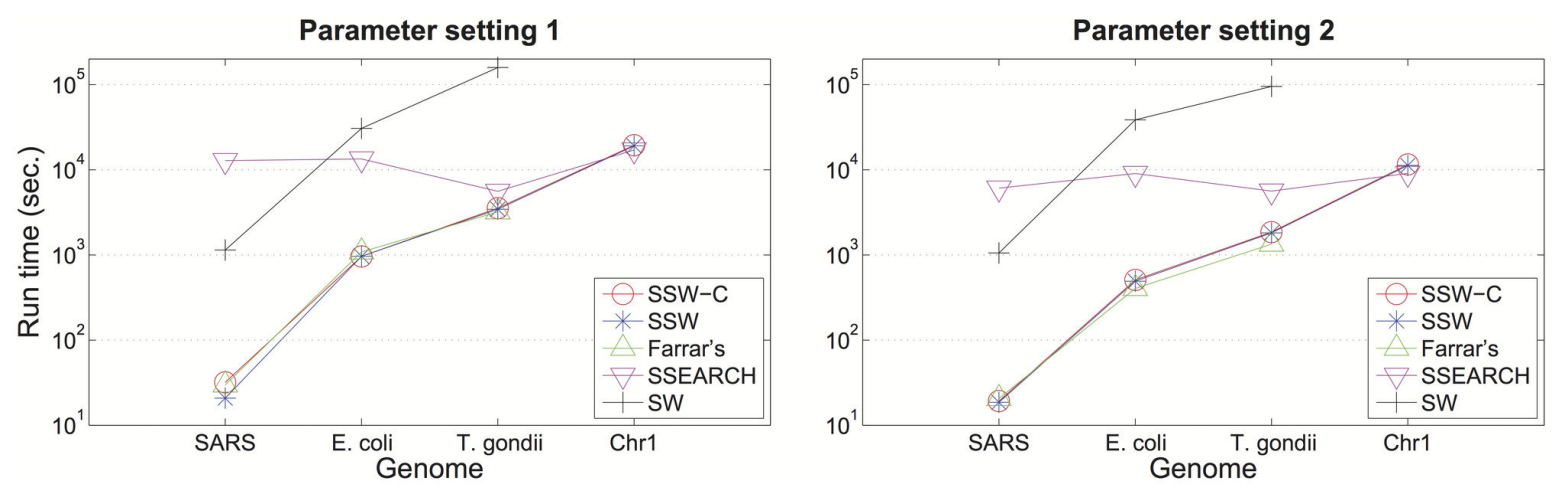
Running time of different SW implementations for real genomic read alignment. Running time of aligning 1,000 real sequencing reads to various microorganism genomes and the human chromosome 1 are shown. Farrar’s implementation cannot handle long sequences as human chromosome 1, so its corresponding running time is not shown here. The log-scaled running time is shown on the y-axis for SSW without (blue) and with (red) detailed alignment, Farrar’s implementation (green), SSEARCH (pink) and an ordinary SW implementation (black). All SW implementations were tested under two sets of SW parameters: scores of match, mismatch, gap open and extension are 2, -1, -2, and -1 respectively (left), and scores of match, mismatch, gap open and extension are 1, -3, -5, and -2 respectively (right).

For the comparisons on real sequencing datasets, we aligned four sets of a thousand reads representing three different sequencing technologies against four different reference genomes: (1) Applied Biosystems (ABI) capillary reads (1,388 bp average length) against the severe acute respiratory syndrome (SARS) virus (29,751 bp); (2) Ion Torrent reads (236 bp) against *E. coli* (4.94 × 10^3^ bp); (3) Illumina reads (100 bp) against *T. gondii* (6.08 × 10^7^ bp); and (4) Illumina reads against human genome chromosome 1 (2.49 × 10^8^ bp) as shown in Figure 3. The same SW parameter settings as the tests on the simulated data sets are used in the experiment. The detailed genome and read information is described in Appendix S1 in File S1. The command lines used for read alignments are shown in Table S2 in File S1. These results indicate that even while returning a full optimal alignment and one suboptimal score, our SSW algorithm is just as fast as Farrar’s accelerated version. 

We note that the relative performance of SSEARCH against our method is worst when working with short target DNA sequences, which is exactly the context in which pairwise alignment is most likely to be used.

### Applications

Here we demonstrate the utility of our SSW library as a component of four different biologically meaningful applications.

#### Primary short-read mapper

To provide highly accurate alignments, most short-read mappers integrate an SW algorithm for a final “polishing” step. This step is especially important for aligning reads containing short insertions and deletions. Even though each SW run is short, it may be applied hundreds of millions of times within a single run of a mapper, and therefore even small inefficiencies result in wasteful resource usage. To quantify time savings with SSW, we compared the performance of our new method with the existing SW implementation within the MOSAIK mapping program [14], which uses SW for the final read alignments. We found that the SSW library achieves a two-fold speedup of the entire MOSAIK compared with the current banded SW implementation within it (see Table 1). Notably, MOSAIK is a multi-threaded program and thus the SSW component in MOSAIK is running in parallel.

**Table 1:** Comparison of the running time (seconds) between the banded SW engined MOSAIK and the SSW engined MOSAIK.

	Illumina 100 bp	454
Banded SW	70145.760	240535.730
SSW	38927.380	98198.990

We aligned three million Illumina 100 bp reads and one million 454 reads against the human genome.

#### Secondary short-read mapper

Primary read mappers are often unable to map or properly align reads in structural variant (SV) regions, e.g. in regions of deletions, insertions, inversions, or translocations. Therefore, we developed a split-read aligner program, SCISSORS (https://github.com/wanpinglee/scissors) to map reads across structural variation event boundaries (breakpoints), rescuing reads not mapped, or inaccurately mapped by primary mapping approaches. We used our SSW library to align “orphaned” or severely “clipped” fragment-end read mates (in the case of read pairs where one end-mate is aligned with high mapping quality, but the other mate is either unmapped or mapped with many unaligned or “clipped-off” bases) to the genomic regions indicated by the well-mapped mates’ coordinates. Inclusion of the SW mapping routines of our SSW library makes accurate and fast split-read alignment for SV detection possible. The split-read mapping functionality, using our SSW library, has also been implemented in the TANGRAM SV detection tool (https://github.com/jiantao/Tangram). TANGRAM is used intensively in the 1000 Genomes Project [15,16] to accurately detect MEIs (mobile element insertions) (http://ftp.1000genomes.ebi.ac.uk/vol1/ftp/technical/working/20120815meicalls/BC/). In SCISSORS and TANGRAM, SSW is also called in multi-threaded way.

#### Read-overlap graph generation

To evaluate evidence for putative SV and large INDEL calls generated by assembly methods, we can employ a read-overlap graph generated by exhaustive pairwise alignment of a set of reads which co-localize in a specific genomic region. We tested the effect of the SSW library in this application against a standard SW implementation (https://github/ekg/smithwaterman). To do the speed comparison test, we generated read-overlap graphs for the genomic region 20:21026000-21027500 using 22,543 reads (70 bp long) from 191 African samples in the 1000 Genomes Project dataset. The sample identifiers are listed in Appendix S2 in File S1. The running time of the read-overlap graph generation program RZMBLR (https://github.com/ekg/rzmblr") embedded with an ordinary SW (1795.66 seconds) is about twice of that embedded with our SSW library (973.02 seconds).

## Conclusions

We developed and made available a fast SW library using SIMD acceleration. By returning not only the optimal alignment score but also the actual alignment, as well as a secondary optimal or suboptimal alignment score, the SSW library is suitable for inclusion into other heuristic genomic sequence analysis programs requiring local SW alignment. The most significant utility of our development, however, is that our algorithms can be readily integrated into C/C++ software without modification of the source code, accelerating development for larger software tools. SSW has already been adopted in four programs developed by our group: the primary read mapping tool MOSAIK, the split-read mapping program SCISSORS, the MEI detector TANGRAM, and the read-overlap graph generation program RZMBLR.

## Methods

**Figure 4:**
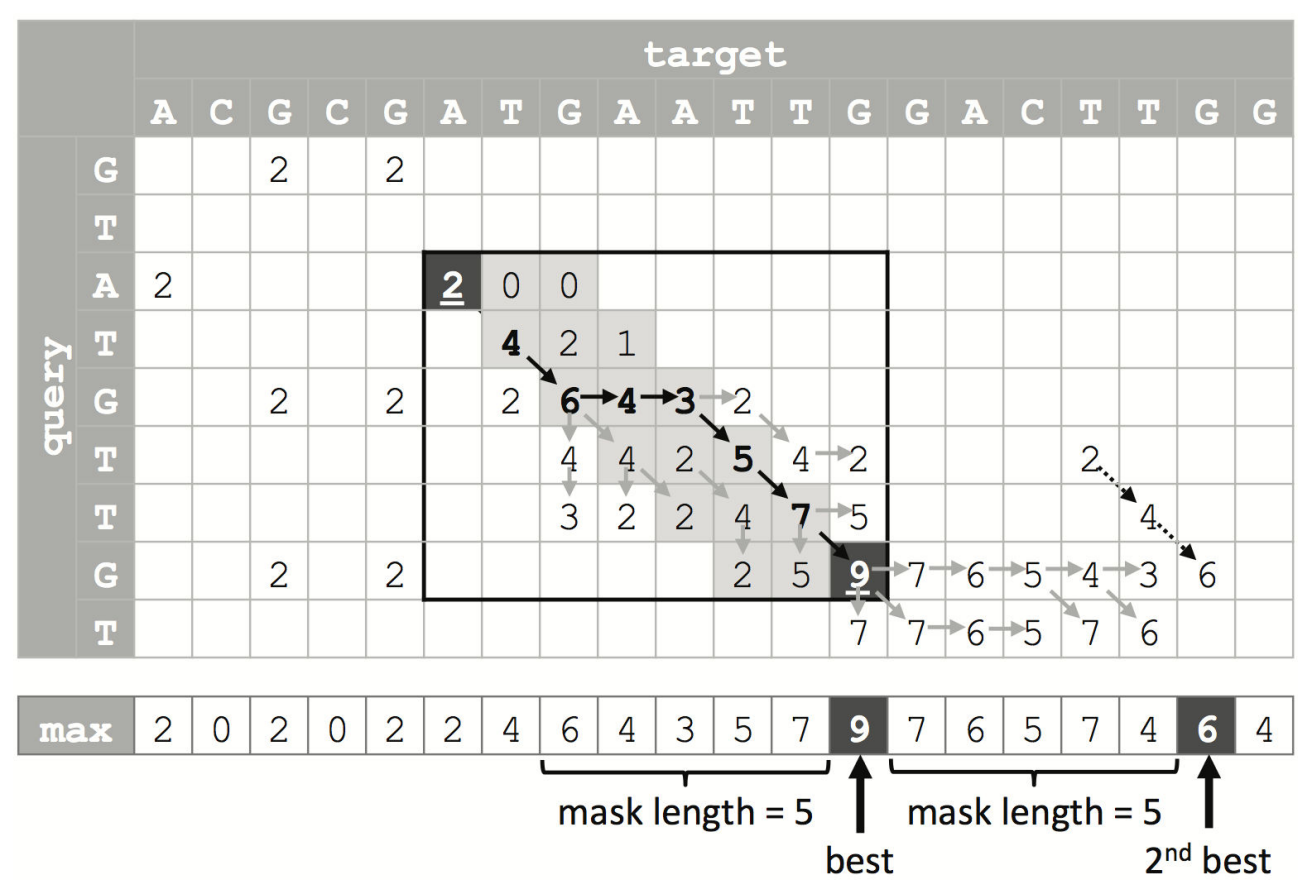
Illustration of alignment traceback and suboptimal alignment score determination. An example SW score matrix is shown (penalties for match, mismatch, gap open and extension are 2, -1, -2, and -1 respectively). The bottom row indicates the maximum score for each column. The algorithm locates the optimal alignment ending position (the black cell with score 9) using the array of maximum scores, and then traces back to the alignment start position (the black cell with score 2) by searching a much smaller, locally computed score matrix (circled by the black rectangle). Finally, a banded SW calculates the detailed alignment by searching the shaded sub-region. The scores connected by solid arrows belong to the optimal alignment. The max array records the largest score of each column. After the optimal alignment score (marked by “best”) is found, its neighborhood is masked, and the second largest score is reported outside the masked region (marked by 2nd best). The scores connected by dashed-line arrows trace the suboptimal alignment.

Our algorithmic improvements focused on speeding up the Farrar’s implementation and gaining access to the optimal alignment (in addition to the optimal achievable score), as well as the score of the best secondary alignment. For speedup, we adopted the “lazy F loop” improvement proposed by SWPS3 [7]. Furthermore we obtained additional alignment information compared to SWPS3 without slowing down the original algorithm: (1) we record the optimal alignment ending positions during the SIMD SW calculation and generate the detailed alignment by a reversed SIMD SW and a banded SW. When the score matrix is filled by the SIMD SW calculation, we store the maximal score of each column in a “max” array and record the complete column that has the maximal score of the whole matrix. Next, we locate the optimal alignment ending position on the reference and the query by seeking the maximal score in the array and the recorded column respectively. The reversed SIMD SW locates the best alignment beginning position from the ending position by calculating a much smaller scoring matrix. Then, the banded SW (whose band is defined by the beginning and ending positions) generates the detailed alignment. Since the alignment generation using the reversed SIMD SW and the banded SW only calculates a very small portion of the whole SW scoring matrix when the query sequence is much shorter than the target, in most cases of genome sequence alignment the corresponding time cost is trivial (2). We determine the secondary alignment score by seeking the second largest score in the “max” array. To avoid a similar sub-alignment of the primary alignment returned, we mask the elements in the region of the primary alignment of the “max” array and locate the second largest score from the unmasked elements (Figure 4). As a crucial step for locating the alignment position and estimating the suboptimal score, the “max” array generation is completed by adding an SSE2 command in the inner loop of Farrar’s implementation and another command in the outer loop, so that this additional time consumption is limited.

## Supporting Information

File S1 (DOCX)
