# Supplementary material for: SSW Library: An SIMD Smith-Waterman C/C++ Library for Use in Genomic Applications
Source: PLoS One. 2013 Dec 4;8(12):e82138. doi: 10.1371/journal.pone.0082138 (PMC3852983; doi:10.1371/journal.pone.0082138)
Supplement: File S1 — (DOCX) [file pone.0082138.s001.docx]

**SSW library: an SIMD Smith-Waterman C/C++ library for use in genomic applications**

**Supporting information**

**Appendix S1. The genomes and reads used for generating Figure 3:**

- SARS: The genome and reads were downloaded from the web site <http://www.bcgsc.ca/project/sars/SARS/>. The SARS reference genome file is TOR2_finished_genome_assembly_290403(Release 3). The 1000 reads were randomly chosen from the file TOR2_genome_shotgun_120403.fasta.
- *E. coli*: The genome is *E. coli* strain 536 downloaded from http://www.ncbi.nlm.nih.gov/nucleotide/NC_008253. The quire sequences are Ion Torrent sequenced *E. coli* strain DH10B (C23-140, 318 PGM Run, 11/2011). Read length: ~25-540 bp. Most reads are ~200 bp.
- *T. gondii*: The genome were downloaded from <http://toxodb.org/common/downloads/release-7.0/TgondiiGT1/fasta/>. The sequences of 14 *T. gondii* chromosomes were extracted from the file [TgondiiGT1Genomic_ToxoDB-7.0.fasta](http://toxodb.org/common/downloads/release-7.0/TgondiiGT1/fasta/TgondiiGT1Genomic_ToxoDB-7.0.fasta) and were used as the reference genome. The Illumina sequenced transcriptome of bone marrow-derived macrophages infected with T. gondii GT1 strain were downloaded from the NCBI Short Read Archive (SRR1002971) and the first 1000 reads in this file were used as the query sequences.
- Chr1: The genome is human reference genome GRCh37. The read file was downloaded from the short read archive (SRR835935). The first 1000 reads were used as the queries.

**Appendix S2. The sample identifiers of AFR in the 1000 Genomes Project:**

HG01879, HG01880, HG01886, HG01896, HG01914, HG01915, HG01958, HG01985, HG01986, HG02013, HG02014, HG02051, HG02449, HG02470, HG02471, HG02489, NA18486, NA18487, NA18488, NA18507, NA18516, NA18520, NA18523, NA18867, NA18868, NA18873, NA18874, NA18908, NA18910, NA18917, NA18923, NA18924, NA18933, NA18934, NA19092, NA19119, NA19130, NA19131, NA19141, NA19143, NA19144, NA19152, NA19153, NA19159, NA19160, NA19171, NA19172, NA19189, NA19197, NA19198, NA19200, NA19204, NA19213, NA19223, NA19235, NA19236, NA19247, NA19248, NA19311, NA19312, NA19313, NA19315, NA19316, NA19317, NA19318, NA19319, NA19321, NA19324, NA19327, NA19328, NA19331, NA19332, NA19334, NA19338, NA19346, NA19347, NA19350, NA19355, NA19359, NA19360, NA19371, NA19372, NA19373, NA19374, NA19375, NA19376, NA19377, NA19379, NA19380, NA19381, NA19382, NA19383, NA19384, NA19385, NA19390, NA19391, NA19393, NA19394, NA19395, NA19396, NA19397, NA19398, NA19399, NA19401, NA19403, NA19404, NA19428, NA19429, NA19430, NA19431, NA19434, NA19435, NA19436, NA19437, NA19438, NA19439, NA19440, NA19443, NA19444, NA19445, NA19446, NA19448, NA19449, NA19451, NA19452, NA19453, NA19455, NA19456, NA19457, NA19461, NA19462, NA19463, NA19466, NA19467, NA19468, NA19469, NA19470, NA19471, NA19472, NA19473, NA19474, NA19625, NA19700, NA19701, NA19703, NA19704, NA19707, NA19711, NA19712, NA19713, NA19818, NA19819, NA19834, NA19835, NA19900, NA19901, NA19904, NA19908, NA19909, NA19914, NA19916, NA19917, NA19920, NA19921, NA19982, NA19985, NA20126, NA20127, NA20276, NA20278, NA20281, NA20282, NA20287, NA20289, NA20291, NA20294, NA20296, NA20299, NA20314, NA20317, NA20322, NA20332, NA20334, NA20336, NA20340, NA20341, NA20342, NA20344, NA20346, NA20348, NA20356

**Table S1. Command lines used for Figure 1:**

| SSW | SSW-C | Farrar | SSEARCH | CUDASW++ 2.0 |
| --- | --- | --- | --- | --- |
| ./ssw_test -p -o12 -e2 database.fasta query.fasta | ./ssw_test -pc -o12 -e2 database.fasta query.fasta | ./farrar blosum50.txt query.fasta database.fasta | ./ssearch36 -p -3 -d1 -T1 query.fasta database.fasta | ./cudasw -db database.fasta -query query.fa -mat blosum50 |

**Table S2. Command lines used for Figure 2 and 3:**

Parameter setting 1:

| SSW | SSW-C | Farrar | SSEARCH | SW |
| --- | --- | --- | --- | --- |
| ./ssw_test –m2 –x1 –o2 –e1 reference.fa reads.fa | ./ssw_test –m2 –x1 –o2 –e1 -c reference.fa reads.fa | ./farrar -i -2 -e -1 matrix.txt reads.fa reference.fa | ./ssearch36 -T1 -d1 -g -1 -f -2 -r +2/-1 -3 -n reads.fa reference.fa | ./sw –m2 –x1 –o2 –e1 reference.fa reads.fa |

Parameter setting 2:

| SSW | SSW-C | Farrar | SSEARCH | SW |
| --- | --- | --- | --- | --- |
| ./ssw_test –m1 –x3 –o5 –e2 reference.fa reads.fa | ./ssw_test –m1 –x3 –o5 –e2 -c reference.fa reads.fa | ./farrar -i -5 -e -2 matrix.txt reads.fa reference.fa | ./ssearch36 -T1 -d1 -g -2 -f -5 -r +1/-3 -3 -n reads.fa reference.fa | ./sw –m1 –x3 –o5 –e2 reference.fa reads.fa |
